# Nonlinear Effects of the Neighborhood Environments on Residents’ Mental Health

**DOI:** 10.3390/ijerph192416602

**Published:** 2022-12-10

**Authors:** Lin Zhang, Suhong Zhou, Lanlan Qi, Yue Deng

**Affiliations:** 1 Institute of Studies for the Greater Bay Area (Guangdong, Hong Kong, Macau), Guangdong University of Foreign Studies, Guangzhou 510006, China; 2 School of Geography and Planning, Sun Yat-sen University, Guangzhou 510006, China; 3 Guangdong Provincial Engineering Research Center for Public Security and Disaster, Guangzhou 510275, China; 4 School of Management, Guangdong Industry Polytechnic, Guangzhou 510300, China; 5 School of Architecture and Civil Engineering, Chengdu University, Chengdu 610106, China

**Keywords:** built and social environments, mental health, nonlinear relationships, thresholds, random forest

## Abstract

In the context of rapid urbanization and the “Healthy China” strategy, neighborhood environments play an important role in improving mental health among urban residents. While an increasing number of studies have explored the linear relationships between neighborhood environments and mental health, much remains to be revealed about the nonlinear health effects of neighborhood environments, the thresholds of various environmental factors, and the optimal environmental exposure levels for residents. To fill these gaps, this paper collected survey data from 1003 adult residents in Guangzhou, China, and measured the built and social environments within the neighborhoods. The random forest model was then employed to examine the nonlinear effects of neighborhood environments on mental health, evaluate the importance of each environmental variable, as well as identify the thresholds and optimal levels of various environmental factors. The results indicated that there are differences in the importance of diverse neighborhood environmental factors affecting mental health, and the more critical environmental factors included greenness, neighborhood communication, and fitness facility density. The nonlinear effects were shown to be universal and varied among neighborhood environmental factors, which could be classified into two categories: (i) higher exposure levels of some environmental factors (e.g., greenness, neighborhood communication, and neighborhood safety) were associated with better mental health; (ii) appropriate exposure levels of some environmental factors (e.g., medical, fitness, and entertainment facilities, and public transport stations) had positive effects on mental health, whereas a much higher or lower exposure level exerted a negative impact. Additionally, this study identified the exact thresholds and optimal exposure levels of neighborhood environmental factors, such as the threshold (22.00%) and optimal exposure level (>22.00%) of greenness and the threshold (3.80 number/km^2^) and optimal exposure level (3.80 number/km^2^) of fitness facility density.

## 1. Introduction

With the rapid development of global urbanization, mental health is not only a major public health problem in countries around the world, but it is also regarded as one of the most pressing and critical development issues in recent periods, resulting in an enormous socioeconomic and health burden. Nowadays, mental health has become a public health priority in the United Nations Sustainable Development Goals (SDGs) and the global development agenda [1]. According to the World Health Organization, there were nearly a billion people in the world with mental disorders in 2019 [2].

In 2018, the White Paper on Mental Health of Chinese Urban Residents reported that 73.6% of urban residents had a poor mental health status and 16.1% of residents suffered from some psychological problem, while only 10.3% of residents had a good mental health status. Hence, it is urgent to solve the increasingly serious mental health crisis and ultimately improve the psychological status of urban inhabitants in their daily lives. However, rapid urbanization and its related environmental hazards (e.g., air and noise pollution, lack of green space, poor built environment, and reduced social cohesion) may harm residents’ mental health and pose a growing challenge to the public health system [3,4,5]. In the context of the “National Environment and Health Action Plan (NEHAP)” and the “Healthy China” strategy, the health benefits or risks of environmental exposures have attracted wide attention from multiple domains. It is necessary to improve people’s mental health by exerting the salutogenic effects of exposure to better environments and mitigating the adverse impacts of environmental threats.

Recently, an increasing number of studies have examined associations between neighborhood environments and people’s mental health. Urban inhabitants’ exposure to well-designed and attractive environments in residential neighborhoods may positively affect their psychological status, such as those with sufficient and favorable green spaces, various facilities, abundant health resources, mixed-use areas, and enough social communication and safety [5,6,7,8,9,10]. Although current studies advance environmental health research, they tend to assume a linear relationship between neighborhood environments and mental health. Actually, their association may be complex and nonlinear. For instance, a much higher or lower level of environmental exposure may lead to a decline in health status, whereas the appropriate environmental exposure level can have positive effects on individual health [11,12]. Moreover, only a limited number of studies have focused on the threshold effects of environmental exposures (e.g., greenness, PM_2.5_ concentrations, noise, and temperature) on health outcomes (e.g., general health, mood), and then identified the exact threshold values and optimal exposure levels of these environmental factors [13,14].

Overall, accumulating literature describes the beneficial or detrimental effects of neighborhood environments on residents’ mental health. Although some studies have attempted to analyze nonlinear associations between environmental exposures and health behaviors and outcomes, they typically use only one dimension of the environment (e.g., built environment) or one environmental element (e.g., greenness) and focus on individual behaviors (e.g., physical activity, walking, and travel behavior), some diseases, general health level, or satisfaction [14,15,16,17,18]. Relevant explorations of the complicated nonlinear relationships between neighborhood environments and people’s mental health, as well as the importance of various environmental variables that affect mental health, are rather limited. To eliminate or reduce environmental health hazards, it is essential to analyze the threshold effects of diverse environmental variables on health, as well as accurately capture the exact thresholds, optimal levels, and danger ranges of environmental exposures for urban residents, thereby helping to control their environmental exposure within optimal levels and avoid dangers when thresholds are reached or exceeded. Nevertheless, less attention has been paid to the thresholds and optimal exposure levels of neighborhood environmental factors. In addition, the pre-specified associations between explanatory variables (i.e., neighborhood environments) and outcomes (i.e., mental health) in the linear regression models are flawed, whereas the random forest regression model is more flexible and precise in investigating nonlinear relationships [19].

**Figure 1:**
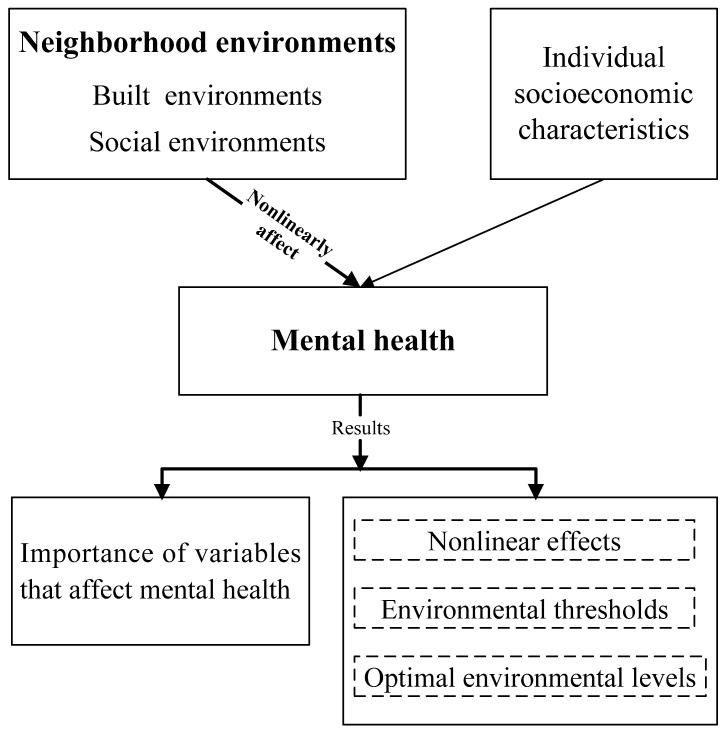
Research framework.

Therefore, this article aims to fill these gaps in the environmental health literature. As shown in Figure 1, this paper first evaluates the importance of neighborhood environments (including built and social environments) in affecting residents’ mental health. The paper then mainly examines the nonlinear or threshold effects of neighborhood environmental factors on mental health and further identifies the accurate thresholds and optimal exposure levels of multiple environmental factors. In addition, the paper explores whether there are significant differences in mental health levels among different populations (including gender, age, marital status, education level, and personal monthly income). This study sheds new light on the nonlinear relationships between neighborhood environments and individual mental health, which will enrich and deepen the understanding of environment–health connections. Furthermore, by ascertaining the exact thresholds and optimal levels of environmental exposures for residents, this paper effectively provides practical implications for more humanistic neighborhood planning, appropriate environmental interventions, and mental health promotion.

## 2. Literature Review

### 2.1. Neighborhood Environments and Residents’ Mental Health

The importance of neighborhood environments (e.g., built and social environments) in influencing residents’ mental health has been widely established in the existing literature [10,20,21,22,23]. To date, many studies have explored greenness within residential neighborhoods and its positive effects on people’s mental health [8,24,25]. The main findings point out that individual exposure to residential greenness in daily life may reduce some environmental threats (e.g., exposure to air pollutants, noise, and high temperature), encourage physical activity, and facilitate neighborhood interaction and social cohesion, thereby restoring capacities, reducing mental disorders, and exerting health-promoting effects [8,26]. Meanwhile, various facilities and services have been associated with residents’ psychological health [6,27,28]. Specifically, enough medical facilities and recreational areas offers numerous benefits to urban dwellers, including better access to medical treatments, abundant health resources, and more opportunities for entertainment activities, thus improving their psychological health [5]. In general, a higher density of fitness facilities is significantly related to better mental health, indicating that ample fitness facilities may alleviate people’s negative psychological status (e.g., perceived stress, anxiety, and depressive mood) through increasing the opportunity and convenience to undertake more physical activity [29]. However, several studies have reported that there is no statistically significant correlation between fitness facility density within their neighborhood and residents’ mental health. For the density of public transport stations, previous research has analyzed its relationship with individual mental health, but the findings are inconsistent. For example, plenty of public transport stations nearby can reduce individual travel distances and improve accessibility to a variety of facilities and resources, thereby mitigating the negative effects of long-distance travel and promoting life satisfaction and mental well-being [29,30]. If the urban built environment is suitable for active transport modes (e.g., cycling), people are likely to have more positive moods (e.g., happiness) and less negative moods (e.g., fatigue, stress, and sadness) during their travel [31]. In contrast, Zhang et al. (2021) revealed that people who live in neighborhoods with a higher density of public transport stations may have a poor mental health [5]. In addition, other built environment factors (e.g., housing conditions, residential density, population density, land use mix, and access to commercial facilities) are likely to affect individual mental health [7,10,32]. Rautio et al. (2018) found that adverse built environments and poor housing quality may have significant correlations with people’s depression [33]. Several studies have analyzed the relationship between population density and individual psychological status (e.g., happiness, annoyance) [34,35,36].

Concerning social environments, some studies have estimated the impacts of social communication on mental health. For instance, Zhang et al. (2019) observed that people who enjoy communication with their families and neighbors, as well as actively engage in group activities and social interaction in their residential neighborhoods, tend to have a positive psychological status [10]. Qiu et al. (2019) suggested that neighborhood interaction and reciprocity are positively associated with residents’ mental health [23]. Social safety is another important factor that affects people’s psychological health. Prior research has found that a higher level of neighborhood safety is strongly related to better mental health [9,37]. Lorenc et al. (2012) reviewed the existing literature and concluded that neighborhoods with low security and high crime rates may induce individual anxiety and insecurity, thus negatively affecting mental health [38]. Moreover, some studies have examined associations between neighborhood cohesion [39] and neighborhood trust [40] with individual mental health, reporting inconsistent results.

### 2.2. Nonlinear Relationships between Neighborhood Environments and Mental Health

The vast majority of existing studies exploring relationships between neighborhood environments and mental health assume linearity, but nonlinear relationships between environmental factors and human health are possible [13,14]. For example, a little empirical evidence has shown nonlinear associations (e.g., inverted U-shaped associations, curvilinear response associations) between exposure to residential greenness and health outcomes (e.g., mortality, pulmonary disease, and general health) [12,16,41]. Such results have verified that people who live in neighborhoods with lower levels of greenspace exposure may have limited opportunities for contact with greenness, as well as increased risk of exposure to environmental hazards (e.g., air and noise pollution, excessive heat) and insufficient physical activity, thereby resulting in health-constraining effects. Also, a much higher level of greenness nearby was associated with people’s exposure to more deleterious environments (e.g., pesticides, herbicides), which may seriously harm their health outcomes (e.g., asthma, allergy, and poor general health) [14,42]. While earlier research has affirmed the beneficial influence of appropriate greenspace exposure on mental health, such as providing a healing setting for stress alleviation and psychological relaxation, promoting diverse physical activities, and improving environmental quality, much remains to be learned about the complex relationships between greenspace exposure and mental health and how these relationships may vary by population group, as well as how to define and quantify “appropriate greenspace exposure.” In addition, existing evidence argues that the proper density of built environments (e.g., various facilities, public transport stations, and road networks) is positively associated with health status. However, high-density urban environments may lead to increased exposure to air and noise pollution, lack of public green space, and unsafe environments, which could directly or indirectly harm health behaviors (e.g., sedentary behavior, insufficient physical activity) and outcomes [5,10,11,43]. As a consequence, these potentially contradictory results may cause a nonlinear relationship between built environment factors and health. In addition, Dong et al. (2021) analyzed the nonlinear psychological effects of social environments in residential communities [44].

Several recent studies have not only detected nonlinear relationships between environmental factors and health behaviors (e.g., walking) and outcomes, but they have also highlighted potential threshold effects [14,45,46]. The threshold effect is a special type of nonlinear relationship, where the change in impact increases or decreases after exceeding a threshold, which is the point or range of an explanatory variable [13,19]. In other words, when exceeding the threshold, a slight change in the explanatory variable (e.g., neighborhood environmental variable) will cause a dramatic change in the outcome (e.g., health) [47,48]. Researchers have found that threshold effects may be manifested in neighborhood attributes, which has benefits and practical implications for urban planners and policymakers in developing more effective environmental interventions [49,50,51,52,53]. For instance, Huang et al. (2022) proved an inverted U-shaped link between residential greenness and general health, with a turning point (i.e., threshold value) at an NDVI value of 0.40 [14]. Zhang et al. (2020) identified the thresholds of micro-environmental variables (e.g., noise, temperature, and relative humidity) in bus cabins and examined the optimal micro-environmental exposure levels for passengers that helped improve their psychological status (e.g., momentary mood) [13]. Additionally, Dong et al. (2019) explored the nonlinear effects of neighborhood characteristics on pedestrian satisfaction [54]. Moreover, it has been confirmed that built environment variables do not have equivalent effects across the entire range of the variable [55]. Therefore, it is important to identify and quantify the optimal level of environmental exposure that exerts the greatest positive effects on people’s health status.

## 3. Method and Data

### 3.1. Method

Random forest regression was used to evaluate the importance of environmental factors on mental health and then explore the complex nonlinear relationships between neighborhood environments and residents’ mental health, after controlling for individual socioeconomic characteristics. 

Random forest is one of the most influential and powerful machine learning algorithms, which has been widely applied in the fields of environmental studies and health [19,56,57,58]. Compared with the linear regression model, which describes a particular linear correlation between explanatory variables and outcome, the random forest approach does not make this pre-specified assumption (i.e., linear relationship) [18,59]. The variable importance measure is a vital output of the random forest regression analysis. That is, the random forest regression model can effectively quantify the importance of explanatory variables in predicting the outcome. Using the percentage of Increased Mean Square Error (%IncMSE) as the evaluation criterion. It is calculated as the average difference of the variables’ mean square error from the original dataset and the sets of randomly permuted variables, which is one of the most efficient approaches for identifying important variables [60,61]). This paper quantified the importance value of each influential factor. The larger the value, the more important the factor. Additionally, the calculated importance value reflects the ability of various influential factors to predict mental health. Moreover, the random forest regression model can excellently capture more refined and complicated associations among variables [62,63,64,65,66]. Specifically, it produces a partial dependence plot for visualizing the direction of links between each predictor and outcome, revealing their nonlinear relationships and illustrating the potential threshold effects [18,67,68,69]. In this paper, the partial dependence plot depicted the marginal effect of a neighborhood environmental factor on individual psychological status while controlling for the average effects of all other explanatory variables in the model. Also, the plot illustrated the nonlinear effects of neighborhood environments on people’s mental health within certain ranges.

A random forest is a tree-based ensemble approach that optimizes model fit, reduces prediction variance, and achieves higher predictive accuracy by combining multiple decision trees [57,70,71]. Two important parameters (the number of trees and number of variables tried at each split) of the random forest can significantly affect the modeling performance and results [72,73,74]. The number of trees refers to the number of decision trees contained in the random forest model. To obtain the optimal specification of the random forest, this study performed the model with the number of trees ranging from 10 to 1000 in increments of 10. Setting the number of trees to 500 can create a more robust model and ensure the better predictive performance of the model [19]. The number of variables tried at each split is recommended to be one-third of the total number of explanatory variables [57]. Accordingly, 4 was found to be optimal for developing the random forest regression model in this research.

### 3.2. Questionnaire Survey

**Figure 2:**
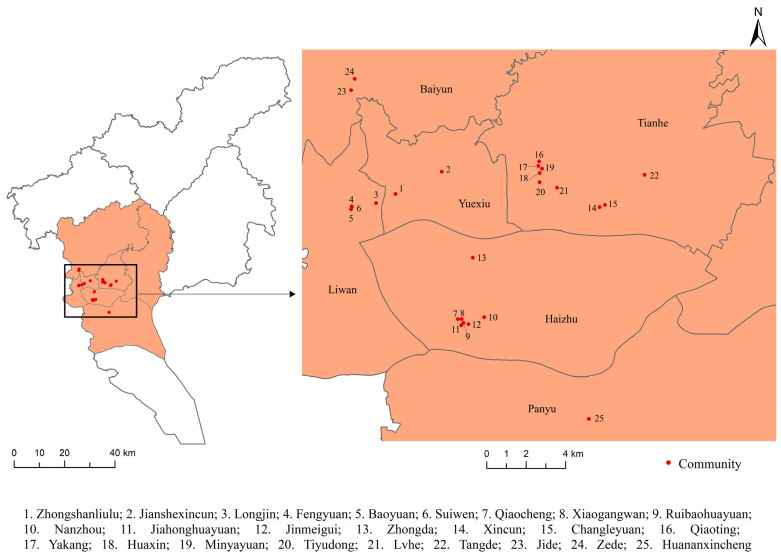
Study area and 25 sampled communities.

A questionnaire survey of urban residents was conducted in August 2017 in the 25 communities of Guangzhou, China. Guangzhou has a total area of 7434.4 km^2^ and had a permanent population of approximately 14.5 million in 2017. The density of the permanent population was approximately 1950 persons/km^2^ in Guangzhou in 2017. These sampled communities were located in the six districts (Liwan, Yuexiu, Haizhu, Tianhe, Panyu, and Baiyun) of Guangzhou (Figure 2). The specific sampling strategy of participants was introduced by Zhang et al. (2021) [5]. Participants were asked to complete the questionnaire, which included detailed information about personal and household attributes, individual health outcomes, and neighborhood contextual issues. After removing questionnaires with incomplete and illogical responses, 1003 questionnaires were obtained from the participants. All participants were fully informed about the study design and provided informed consent.

### 3.3. Data Description

#### 3.3.1. Neighborhood Environments

Drawing upon prior literature [5,6,9,10], the neighborhood environments that affect people’s mental health may include built and social environments. In this paper, a 500 m radius buffer was chosen based on the urban features of the study area and the spatial scale/range of participants’ activity within their neighborhoods. We also collected information from some participants regarding how long (about 5–10 minutes) and how far (approximately 500 m) they were willing to walk to reach facilities and services in the study area. Additionally, previous studies have reported that neighborhood built environments can be effectively captured using a 500 m radius buffer based on the participants’ residential locations [5,75,76]). The 500 m radius buffer around each participant’s residential location was used to delineate contextual areas for extracting and measuring the built environment variables. More specifically, greenness was the proportion of the area of green space within the residential buffer [Greenness (%) = Area of green space (km^2^)/Area of residential buffer (km^2^)]. It was extracted and measured from Landsat-8 satellite images covering the study area. The density of various facilities (medical, fitness, and entertainment facilities, and public transport stations) was used to represent the compactness of social and economic activities and the diversity of urban functions, as well as reflect the convenience of residents to undertake these activities [5,34]. Specifically, the density of medical facilities was used to represent residents’ accessibility to medical facilities. The density of fitness and entertainment facilities was used to reflect the convenience and opportunity of people to engage in these activities. Also, the density of public transport stations (e.g., bus stations, subway stations) was used to represent the accessibility and convenience of public transportation. The facility density was the proportion of the number of a facility within the residential buffer [Facility density (number/km^2^) = The number of a facility (number)/Area of residential buffer (km^2^)]. It was extracted and calculated from the Points-of-Interest (POIs) data of the study area.

In addition, the social environment includes neighborhood communication and neighborhood safety, which were collected in the questionnaire. The residents’ perceptions or assessments of neighborhood communication and neighborhood safety were respectively rated from “extremely poor” to “extremely good” with a score that increased from 1 to 5.

#### 3.3.2. Mental Health

Mental health was measured using the World Health Organization’s Five Well-Being Indexes (WHO-5), which has been universally used in relevant research [77]. The five items of the WHO-5 included (i) I have felt cheerful and in good spirits; (ii) I have felt calm and relaxed; (iii) I have felt active and vigorous; (iv) I woke up feeling fresh and rested; (v) My daily life has been filled with things that interested me [78]. Additionally, its reliability and validity have been confirmed to be excellent [5,78]. Participants were requested to answer each item of the WHO-5 on a 5-point scale from 1 (never) to 5 (always). The total scores of mental health spanned between 5 and 25, and participants with a higher score had a healthier psychological status.

#### 3.3.3. Individual Socioeconomic Characteristics

The average age of the participants was nearly 36, half were male, more than 60% achieved a college or higher education degree, and approximately 70% of the participants had a personal monthly income below the average level (7210 Yuan) (data from the Annual Salary Survey Report of Guangdong Province in 2017) in Guangzhou in 2017. The descriptive statistics of all variables are summarized in Table 1.

## 4. Results

### 4.1. Importance of Variables That Affect Mental Health

The overall explanatory power of this random forest regression model was 39.37%. Table 2 intuitively presents %IncMSE and the ranking of variable importance. The results indicated that neighborhood environments (built and social environments) contribute the most to the predictive power of the random forest regression and individual socioeconomic characteristics are indispensable in affecting mental health. Specifically, greenness was the most important factor influencing mental health, with an importance of 36.23%. Additionally, greenness had a greater impact on individual mental health than other environmental factors and individual socioeconomic characteristics. The importance of neighborhood communication (34.80%) and fitness facility density (31.59%) on mental health ranked second and third, respectively, which suggested that these two variables strongly affect mental health, followed by entertainment facility density in fourth place (30.44% importance), public transport station density in fifth place (26.17% importance), and medical facility density in sixth place (22.13% importance). Notably, neighborhood safety was ranked relatively low, being in ninth place with an importance of 14.32%. This finding revealed that neighborhood safety may not significantly influence mental health since the perceived neighborhood safety of most participants was already high in this study (the mean score of neighborhood safety was 3.98 and the standard deviation was 0.84). Among individual socioeconomic characteristics, people’s education level (19.10% importance) exerted a greater effect on mental health, followed by marital status in eighth place (14.48% importance), age in tenth place (11.42% importance), and gender in eleventh place (2.75% importance). Personal monthly income was the least important factor affecting mental health, accounting for only 1.91%. Such a result was relative and partially related to the income structure of the participants in this study.

### 4.2. Nonlinear Effects of Neighborhood Environments on Mental Health

**Figure 3:**
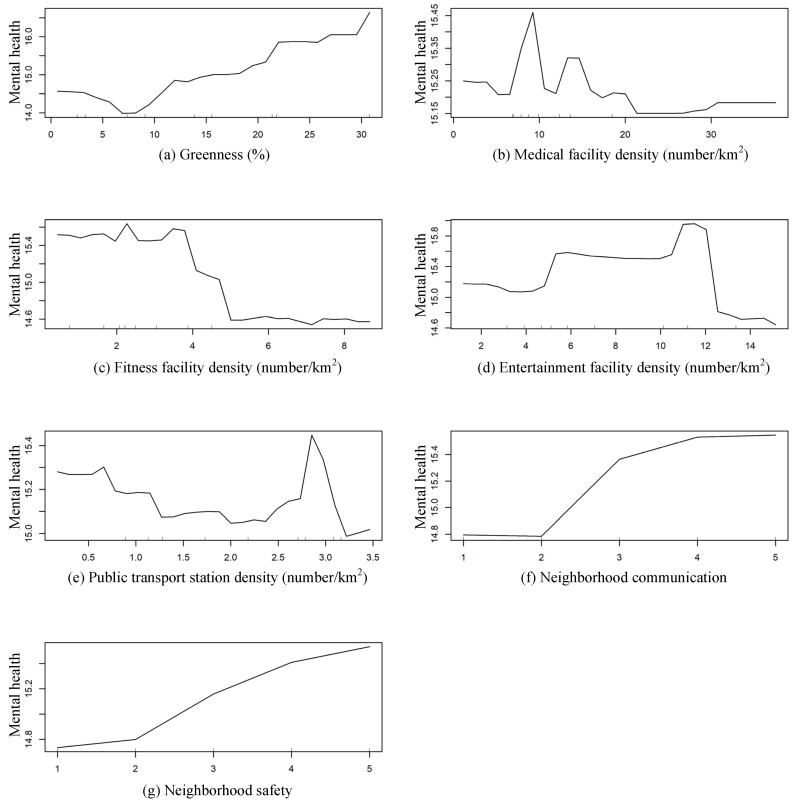
Nonlinear effects of neighborhood environments on mental health.

The partial dependence plot provided a fine-grained analysis of the complicated nonlinear associations between neighborhood environments and people’s mental health (Figure 3). The nonlinear effects were observed to be universal and varied among neighborhood environmental variables, which could be classified into two categories:

(i) The internal elements of the neighborhood, including greenness, neighborhood communication, and neighborhood safety, may be easily accessed or perceived by residents. Specifically, as shown in Figure 3a, greenness between 7.00% and 22.00% was positively linked to individual mental health (i.e., mental health level improved with an increase in greenness). Beyond this critical value of greenness (22.00%), people’s exposure to more greenness was likely to create a more stable and better mental health. Therefore, 22.00% was found to be the threshold of the impact of greenness on mental health, suggesting that greenness in neighborhoods should be greater than 22.00%. A score of neighborhood communication below or equal to 2 may not significantly and positively affect individual mental health, and mental health remained at a lower level (Figure 3f). Only when the score was above 2, a positive association between neighborhood communication and mental health could be clearly observed. Thus, 2 was found to be the threshold for the score of neighborhood communication beyond which mental health got better with an increase in the score. An explanation for this may be that residents who enjoy communication and interactions with other people in residential neighborhoods are much more likely to have better mental health. In other words, closer neighborhood communication, richer social networks, and more availability in neighborhoods can contribute to improving people’s mental health and reducing psychological problems. Moreover, there was an overall upward tendency in the partial dependence plot (Figure 3g), demonstrating that people who undertake daily activities and spend some time in safer and more attractive neighborhoods tend to have better psychological health.

(ii) The external elements (i.e., supporting facilities) of the neighborhood include medical, fitness, and entertainment facilities, and public transport stations. To be specific, the nonlinear effects of medical facility density on individual mental health can be seen in Figure 3b. Medical facility density seemed to be positively related to mental health if the medical facility density ranged from 1.17 to 9.00 number/km^2^, with a peak positive effect at 9.00 number/km^2^. This result indicated that adequate medical facilities and higher accessibility to them can meet people’s living needs of getting medical treatment quickly and easily in their neighborhoods, thereby greatly enhancing mental health. When exceeding the threshold value (9.00 number/km^2^), an increase in the density of medical facilities was associated with a decreasing trend in mental health level, suggesting that excessive medical facilities may have negative impacts on individual psychological status. Overall, the optimal density of medical facilities for residents was 9.00 number/km^2^ within the residential buffers, and too many or too few medical facilities negatively influenced mental health. In terms of fitness facility density (Figure 3c), people’s mental health remained healthier and more stable within the interval of 2.00 and 3.80 number/km^2^. However, when this threshold value (3.80 number/km^2^) was exceeded, the mental health level declined rapidly and reached the lowest level at approximately 8.66 number/km^2^. Although convenient access to surrounding fitness facilities can support residents’ physical activity, promote pleasant experiences, and alleviate the risk of mental disorders (e.g., stress, anxiety, and tension), too many fitness facilities may compress the space for other types of facilities and reduce the diversity of land use. Hence, approximately 3.80 number/km^2^ was the proper density of fitness facilities within neighborhoods. Additionally, Figure 3d showed the nonlinear relationship between the entertainment facility density and people’s psychological health. An inverted U-shaped curve was observed, with peak mental health effects achieved at the density of 10.00–12.00 number/km^2^. This result implied that residents who lived in neighborhoods with the appropriate density of entertainment facilities (10.00–12.00 number/km^2^) tended to have a better mental health. However, increasing the density of entertainment facilities beyond the threshold value (12.00 number/km^2^) was correlated with a decreasing trend in mental health level. Further, Figure 3e presents the impact of the density of public transport stations on residents’ mental health. Greater mental health effects were observed at a density of public transport stations of approximately 2.70 number/km^2^. When the density exceeded this threshold value (2.70 number/km^2^), people’s mental health level fell as the density increased. This result demonstrated that further increasing the density of public transport stations in highly dense neighborhood environments may induce more risks and negative effects (e.g., traffic-related air pollution and noise, crowding, and lack of physical activity) on residents’ mental health [79,80].

### 4.3. Population Differentiation in Mental Health Level

This study investigated the presence of significant differences in mental health level among different populations using the independent samples *t*-test (gender, age, and marital status) and one-way ANOVA (education level and personal monthly income), respectively (Table 3). The findings indicated that the mental health of young people was considerably better than that of middle-aged people. This may be because young people have fewer psychological problems and are better at alleviating negative moods and adjusting their mental health status in a variety of ways in their daily lives (e.g., physical activities, entertainment activities, and communication with others). The mental health level of single people was higher than that of married people. This may be because single people do not have the excessive pressures and burdens of family life, thereby inducing a more relaxed and healthier psychological status. In addition, there were significant differences in the mental health of people with different educational levels. The higher the education level, the better the psychological health. Such a finding may be because people with a higher education level tend to have a relatively higher socioeconomic status and easier access to health resources, thereby contributing to enhanced mental health. They may also have a richer knowledge of health care and more fitness awareness, which help to relieve mental disorders and promote psychological health. Nevertheless, there were no statistically significant differences in mental health among residents of different genders and personal monthly incomes.

## 5. Discussion

### 5.1. Theoretical Contributions

Compared with most prior studies that have paid attention to the linear relationships between environments and health outcomes, this paper provides meaningful theoretical contributions to the limited body of literature exploring the complicated nonlinear relationships between neighborhood environments (built and social environments) and residents’ mental health. The research findings indicate that there are differences in the importance of various environmental variables in affecting mental health. Additionally, nonlinear associations are proven to be prevalent, with different environmental factors having different nonlinear effects on individual psychological status, which challenges the assumption of linear relationships in past research. Another unique contribution of the present study involves investigating the threshold effects of neighborhood environments on mental health, as well as ascertaining the accurate thresholds and optimal exposure levels of each environmental factor. These comprise the innovation of this research, as they have not been clearly and adequately examined in earlier literature. In addition, the random forest method is employed to effectively reveal the complex nonlinear relationships and further evaluate the environmental threshold effects. In general, this study not only enriches the knowledge of environment–health connections, but also emphasizes the significance of environmental threshold research. Our novel findings and main methodology can provide new insight for a range of areas of interest to urban researchers and geographers.

### 5.2. Practical Implications

Urban neighborhood planning, environmental improvement, and health promotion are important and urgent issues in the context of rapid urbanization and the “Healthy China” strategy. Our research findings have valuable practical implications for planners and policymakers for building healthy and humanistic neighborhoods, creating more pleasant environments, and optimizing public services and facilities, thus alleviating residents’ mental disorders and improving their psychological status in their daily lives. In detail, the lower bound threshold effects are found for greenness in neighborhoods, which suggests that greenness would not exert pronounced and stable positive impacts on mental health until it exceeds 22.00%. Consequently, strategies for designing and planning residential greenness should fully consider the threshold effects observed in this paper, namely, the optimal setting of greenness should be greater than 22.00%. Meanwhile, residents should be advised to increase their utilization of green spaces and promote awareness of environmental protection, and policymakers should scientifically formulate and implement specific measures of greenspace protection and management [78]. Moreover, the optimal levels of medical and entertainment facility density within neighborhoods to effectively enhance residents’ mental health are 9.00 and 10.00–12.00 number/km^2^, respectively. Residential neighborhoods with too many or too few medical and entertainment facilities may induce negative effects on psychological health. The optimal densities of fitness facilities and public transport stations are nearly 3.80 and 2.70 number/km^2^, respectively. Thus, there is a need to take the upper bound threshold effects into account during neighborhood planning and built environment design [18,19], with fitness facilities and public transport stations not being too numerous [5,79,80]. These recommended values of optimal environmental exposure levels are important for developing residential environments and health guidelines. When exceeding the optimal exposure levels of neighborhood environmental factors, planners and policymakers should be adequately aware of the environmental health risks and effectively respond to them, thereby avoiding the dangers of environmental exposures for urban residents and reducing environmental health hazards. Furthermore, better neighborhood communication and safer environments may have positive impacts on residents’ mental health.

In addition, appropriate environmental interventions can effectively influence residents’ mental health. Notably, not all environmental interventions can be implemented with limited public resources and there is commonly a priority ranking among them [19]. In this paper, the evaluation of variable importance identifies the more critical environmental variables within neighborhoods that affect psychological health, further implying the priority of environmental interventions. Therefore, preferentially intervening and improving these more important neighborhood characteristics can help to significantly enhance the psychological status of residents and reduce mental disorders. Specifically, greenness plays the most crucial role in influencing mental health. It thus should be the primary consideration in neighborhood planning and environmental management. This is followed by neighborhood communication and then fitness facilities, which strongly impact individual mental health. These findings may support effective improvement measures and guided policies in terms of strengthening communication and interaction and optimizing fitness facility density when designing environmentally friendly communities. Further, the density of entertainment facilities, public transport stations, and medical facilities is related to residents’ mental health. These factors should be considered when building more comfortable and convenient neighborhood environments.

In summary, it is vital to reveal the salient nonlinear effects of neighborhood environments on mental health. The research findings yield important insights for designing appropriate environmental interventions, optimal environmental exposure levels, and more effective policies, thereby exerting beneficial influences of neighborhood environments and improving people’s psychological status. However, ignoring nonlinear associations or threshold effects may lead to ineffective environmental interventions and even misguided policies [15,17].

### 5.3. Limitations and Future Research

This study has several limitations that should be addressed in future work. First, the cross-sectional design of this research not only impedes causal inference, but also neglects the cumulative threshold effects of neighborhood environments on residents’ mental health over long periods, which may cause some changes in the thresholds and optimal exposure levels of environmental factors. Hence, conducting a longitudinal survey in the future will support more robust evidence on these findings. Second, residents’ subjective assessment of built environment variables should be simultaneously considered in future explorations of the nonlinear relationships between neighborhood environments and mental health. Third, a problem of neighborhood self-selection bias may influence our analysis, since a resident with several unmeasured attributes (e.g., preference for outdoor environments, preference for communicating with others, and ability to undertake more activities) is more likely to live in a neighborhood with enough green spaces and facilities and thus report better mental health. Future work will try to address this limitation by assessing more characteristics (such as preferences, attitudes, and abilities for environments and activities). Fourth, due to outliers, noise, sparse data, and/or spatially uneven distribution of data for the neighborhood environmental variables, the random forest approach used in this study produces fluctuating results for some ranges of the environmental variables. Thus, increasing the amount and density of data in future work may reduce or avoid these fluctuating results and further enhance the performance of random forest regression models [19].

## 6. Conclusions

This paper mainly explored the nonlinear effects of neighborhood environments (built and social environments) on residents’ mental health and evaluated the relative importance of each environmental variable. The paper also identified the accurate thresholds of various environmental factors and optimal environmental exposure levels for residents. According to the results of this study, the importance of neighborhood environments relative to individual socioeconomic characteristics was overwhelming. To be specific, greenness was the most crucial variable affecting residents’ mental health, followed by neighborhood communication, fitness facility density, and the density of entertainment facilities, public transport stations, and medical facilities. Conversely, the importance of neighborhood safety in influencing mental health was relatively low. The neighborhood environmental variables tended to exert significant nonlinear effects on mental health, which could be classified into two categories: (i) higher exposure levels of some environmental factors promoted better mental health. These environmental factors were internal elements of the neighborhood, which could be easily accessed or perceived by each resident, such as greenness, neighborhood communication, and neighborhood safety; (ii) appropriate exposure levels of some environmental factors had positive impacts on mental health, but much higher or lower exposure levels caused a decline in mental health level. These environmental variables were external elements (i.e., supporting facilities) of the neighborhood, including medical, fitness, and entertainment facilities, and public transport stations. In addition, the exact thresholds and optimal levels of environmental exposure identified in this research can provide important insights for building healthy and humanistic neighborhoods and creating attractive environments that improve individual mental health. More explicitly, 22.00% was identified as the threshold of the influence of greenness on mental health, with an optimal level of greenness at greater than 22.00%. The threshold densities for medical facilities, fitness facilities, entertainment facilities, and public transport stations were estimated to be 9.00, 3.80, 12.00, and 2.70 number/km^2^, respectively. Thus, the recommended values of these indicators within neighborhoods are 9.00, 3.80, 10.00–12.00, and 2.70 number/km^2^, respectively. For residents, closer neighborhood communication and safer environments contributed to enhancing their mental health.

## Figures and Tables

**Table 1:** Descriptive statistics for all variables (N = 1003).

Variable		Mean/Percentage	Standard Deviation (SD)
**Mental health**		15.30	3.54
**Neighborhood environments**			
Greenness		13.99%	9.59
Medical facility density (number/km^2^)		10.16	4.75
Fitness facility density (number/km^2^)		2.71	1.86
Entertainment facility density (number/km^2^)		7.21	3.90
Public transport station density (number/km^2^)		2.00	0.96
Neighborhood communication		3.42	0.66
Neighborhood safety		3.98	0.84
**Individual socioeconomic characteristics**			
Gender	Female	50.05%	0.50
	Male	49.95%	
Age	Young people (19–44)	75.37%	0.43
	Middle-aged people (45–59)	24.63%	
Marital status	Single	19.94%	0.40
	Married	80.06%	
Education level	Primary school or lower	0.10%	0.62
	Junior high school degree	6.28%	
	Senior high school degree	27.52%	
	Bachelor degree	65.20%	
	Master degree or higher	0.90%	
Personal monthly income (Yuan)	≤2999	1.20%	0.93
	3000–4999	32.10%	
	5000–8999	48.55%	
	9000–11,999	7.48%	
	≥12,000	10.67%	

**Table 2:** Importance of variables that affect mental health.

Variable	%IncMSE	Rank
**Neighborhood environments**		
Greenness	36.23	1
Medical facility density	22.13	6
Fitness facility density	31.59	3
Entertainment facility density	30.44	4
Public transport station density	26.17	5
Neighborhood communication	34.80	2
Neighborhood safety	14.32	9
**Individual socioeconomic characteristics**		
Gender	2.75	11
Age	11.42	10
Marital status	14.48	8
Education level	19.10	7
Personal monthly income	1.91	12

**Table 3:** Results of independent samples *t*-test/one-way ANOVA of mental health based on individual socioeconomic characteristics.

Individual Socioeconomic Characteristics		Mean	T Value	F Value
Gender	Female	15.29	1.692	
	Male	15.30		
Age	Young people (19–44)	15.44	4.191 **	
	Middle-aged people (45–59)	14.84		
Marital status	Single	16.12	30.647 ***	
	Married	15.09		
Education level	Primary school or lower	15.00		
	Junior high school degree	15.23		
	Senior high school degree	14.60		5.635 ***
	Bachelor degree	15.55		
	Master degree or higher	18.67		
Personal monthly income (Yuan)	≤2999	14.50		
	3000–4999	15.45		
	5000–8999	15.28		0.540
	9000–11,999	15.29		
	≥12,000	14.96		

** and *** represent significance at 5% and 1%, respectively.
